# A consideration of CYP2D6 genetic variations in the Ghanaian population as a potential ‘culprit’ for the tramadol ‘abuse crisis’

**DOI:** 10.1186/s12920-023-01773-8

**Published:** 2024-01-22

**Authors:** Nicholas Ekow Thomford, Susanna Aba Abraham, Samuel Badu Nyarko, Robert Peter Biney

**Affiliations:** 1 Department of Medical Biochemistry, School of Medical Sciences, College of Health and Allied Sciences, University of Cape Coast, Cape Coast, Ghana; 2 Pharmacogenomics and Genomic Medicine Group, School of Medical Sciences, College of Health and Allied Sciences, University of Cape Coast, Cape Coast, Ghana; 3 Division of Human Genetics, Department of Pathology, Faculty of Health Sciences, University of Cape Town, Anzio Road, Observatory, Cape Town, 7925 South Africa; 4 Department of Adult Health, School of Nursing and Midwifery, College of Health and Allied Sciences, University of Cape Coast, Cape Coast, Ghana; 5 Department of Pharmacotherapeutics and Pharmacy Practice, School of Pharmacy and Pharmaceutical Sciences Sciences, College of Health and Allied Sciences, University of Cape Coast, Cape Coast, Ghana

**Keywords:** CYP2D6, Tramadol, Opioid analgesics, Pharmacogenomics, Metabolism, Alleles

## Abstract

**Background:**

Cytochrome P450 2D6 is involved in the metabolism of several important medicines including opioids. Variations in CYP2D6 have been implicated in drug response and according to the Clinical Pharmacogenetics Implementation Consortium Guideline (CPIC) for CYP2D6, dosing for CYP2D6 substrates should be based on variants carried by individuals. Although *CYP2D6* variations in Ghana had been previously recorded, not all variants have been reported in the Ghanaian population. In this exploratory study we set to investigate certain unreported variations in the Ghanaian population in addition to the previously reported ones and use that to understand the tramadol ‘abuse’ crisis that is currently being experienced in Ghana.

**Methods:**

This study employed a convenience sampling approach to include 106 unrelated participants who were recruited as part of the PHARMABIOME project. We successfully genotyped 106 samples using Iplex GOLD SNP genotyping protocol after extracting DNA from these individuals. Allele and diplotype frequencies were undertaken by counting from observed genotypes. Comparison of alleles reported from various studies were done.

**Results:**

Unreported alleles such as *3, *9 and *41 which are classified as no function and decreased function were observed in our study cohort. In addition, variants such as (*1, *2, *4, *5, *10, *17 and *29 were observed with different frequencies. Our study showed 26% representation of intermediate metabolizers (IM) and 2% poor metabolizers (PM) in the study population.

**Conclusion:**

The implications for informal sector workers who use tramadol for recreational purposes, is that IMs and PMs will overdose as they may have reduced analgesic effects which will translate into increased risks of unforeseen adverse events. We therefore propose that *CYP2D6* should be considered in opioid dosage while making use of these observed variations to implement new approaches to tackle the tramadol ‘abuse crisis’ in Ghana.

**Supplementary Information:**

The online version contains supplementary material available at 10.1186/s12920-023-01773-8.

## Introduction

Cytochrome P450 2D6 enzyme is the most highly characterized polymorphic drug-metabolizing enzyme comprising a relatively small percentage (2-6%) of the total cytochrome P450. CYP2D6 plays an important role in the metabolism of approximately 25% of all clinically used medications including opioids [1, 2]. The catalytic activity of CYP2D6 is significantly affected by genetic variations [1, 3]. Several metabolism probe-based studies have elucidated different phenotypes of this enzyme [4–6]. Evidence from these CYP2D6 studies have therefore led to phenotype classifications such as poor metabolizers (PM) when they generally carry two non-functional alleles, intermediate metabolizers (IM) with one functional and one non-functional allele, normal metabolizers (NM) with two functional alleles and ultrarapid metabolizers (UM) with chromosomal rearrangements leading to multiple copies of functional alleles [7]. These potentially explain the large interindividual variations that are encountered in drugs that require CYP2D6 for metabolism.

Pain is the commonest symptoms that causes patients to seek medical care [8] and opioid medications are a fundamental part of the management of moderate to severe pain [9]. In Ghana, although opioids are prescription-only medications, inadequate systemic controls from the Food and Drugs Authority (FDA) and Pharmaceutical Society of Ghana (PSG) enable some community pharmacies and other over-the-counter medicine sellers to dispense opioids for pain without appropriate prescriptions. This inadequate control of access to pain medications poses increased risk for adverse drug events, inadequate pain control and/ or developing opioid misuse syndrome [10]. The analgesic efficacy of a drug describes how the drug is able to carry out its pain relief activity. For opioids, their pain-relieving effects is affected by pharmacogenomic associations [11, 12] which makes certain groups of individuals have either sub-optimal analgesic effects or adverse drug events from normal therapeutic doses.

Commonly used opioids such as codeine [5, 13], hydrocodone [14, 15], oxycodone [4] and tramadol [16–18] are metabolised by CYP2D6 enzyme. Although these opioids may also be metabolised by other enzymes such as CYP3A4/5, CYP2D6 is rather responsible for the important role of the conversion of these medications to their active metabolites such as in the case of codeine and tramadol to morphine and O-desmethyltramadol respectively. CYP2D6 is primarily responsible for conversion of parent compound to 5 -30%active metabolites such as in the case of codeine, tramadol, hydrocodone and oxycodone [11, 19] Thus, alterations in CYP2D6 gravely influences the therapeutic outcomes of pain management using codeine, hydrocodone and tramadol.

Over the past few years, there have been several media reports on tramadol ‘abuse crisis’ in Ghana [20]. This is a common phenomenon among young adults in the West African region and can also be linked to the reported global opioid crisis [21]. The apparent abuse is attributed to tramadol’s euphoric potential and perceived sexual enhancement properties. Thus, tramadol gaining notoriety among Ghanaian youth despite the adverse events associated with its recreational use. Considering the significant effect of CYP2D6 variations in the metabolism of opioids such tramadol, the consequences on pain management, and the reported tramadol ‘abuse crisis’, we sought to identify and describe alleles of *CYP2D6* that are represented in the Ghanaian population that may have consequential effects.

## Materials and methods

### Sample collection

One hundred and six unrelated individuals of different ethnic groups (Akan, Larteh, Ewe, Ga, Dagomba) were recruited from the PHARMABIOME project. These were patients visiting the Ewim Polyclinic, Cape Coast Metropolitan Hospital and the Cape Coast Teaching Hospital all within the Cape Coast Metropolis. The study population was made of black Africans. All participants provided both written and oral informed consent before samples were taken. Five (5) milliliters of blood samples were collected from these participants in EDTA tubes. Ethics for this study was obtained from the Cape Coast Teaching Hospital Ethics Review Committee (CCTHERC/EC/2020/2020/109). All experiments were conducted in accordance with ethical guidelines and regulations.

### DNA extraction and genotyping

DNA was extracted using the E.Z.N. A® blood DNA mini kit (Omega Bio-tek, Inc. Norcross, USA) according to the manufacturer’s guidelines. Genotyping for *CYP2D6* variations, *rs16947* (2851 C > T), *rs1135840* (g.9200G > C), *rs35742686* (g.7569del), *rs3892097* (g.42,128,945 C > T), *rs1065852* (g.42130692G > A), *rs5030655* (g.6727del), *rs5030867* (g.42127856T > G), *rs5030865* (g.42,129,033 C > A), *rs28371706* (g.42129770G > A), *rs59421388* (g.42,127,608 C > T) and *rs28371725* (g.42,127,803 C > T) genotyped using Iplex GOLD SNP genotyping protocol on the Agena MassARRAY® system (Agena Bioscience™, San Diego, CA, USA). The *CYP2D6* alleles (*1, *2, *3, *4, *5, *9, *10, *17, *29, *41) and diplotypes were established from the SNP combinations.

### Statistics

Allele and diplotypes frequencies were computed by counting from observed genotypes. Comparison of allele frequencies between different populations was undertaken.

## Results

**Fig. 1:**
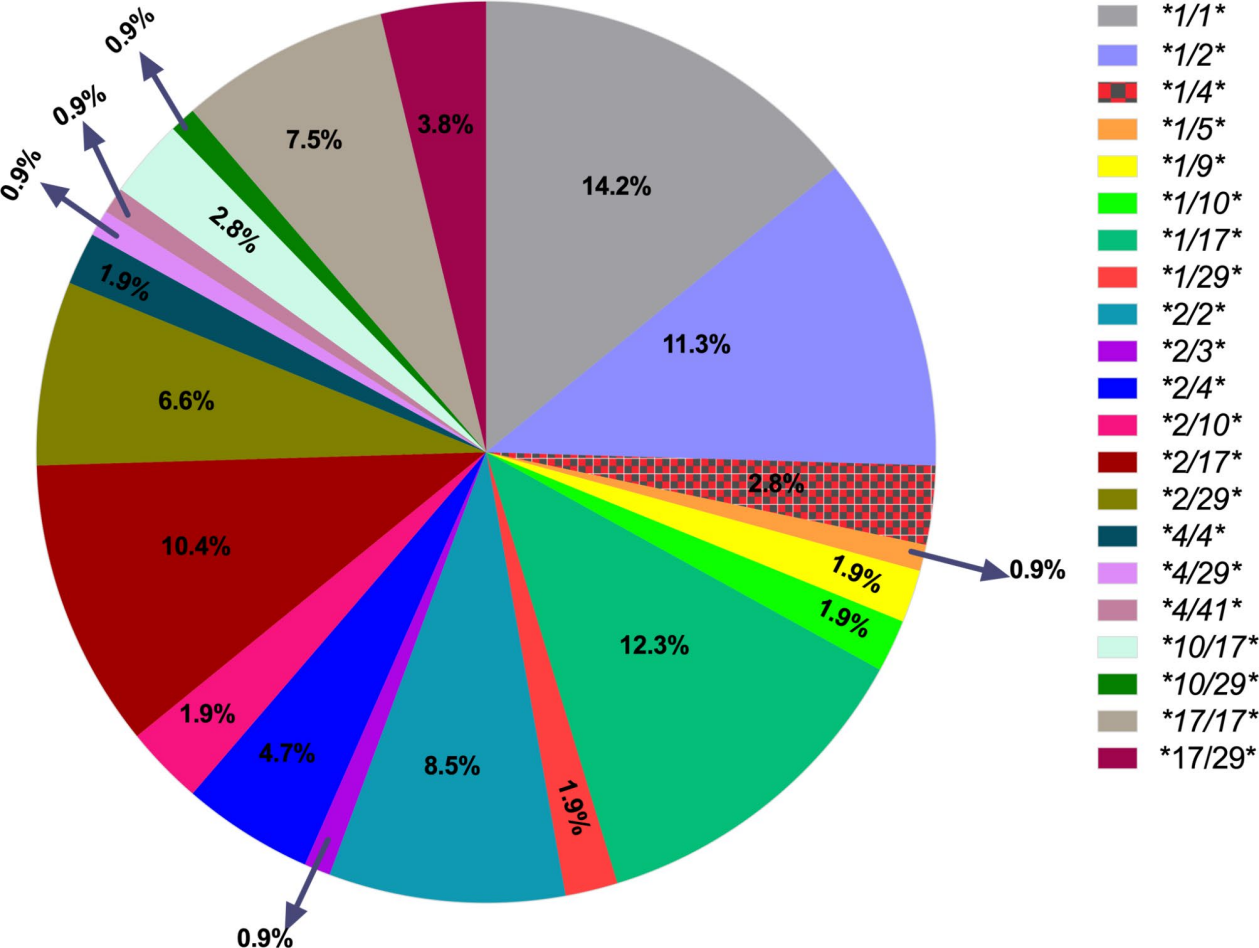
Distribution of *CYP2D6* diplotype frequencies observed in the study population (n = 106). The different colour codes represent the various diplotypes which are translated into the phenotypes according to the CPIC guidelines

The recorded diplotypes and calculated activity scores are shown in Table 1. The frequency of normal metabolizer (NM), intermediate metabolizer (IM) and poor metabolizer (PM) in our study population were 0.72, 0.26 and 0.20 respectively. The respective frequencies of the activity scores were 2 (0.34), 1.5 (0.33), 1.25 (0.05), 1 (0.21), 0.75 (0.04), 0.5 (0.02) and 0 (0.02). *CYP2D6* diplotypes *1/*1, *1/*2, *1/*17, *2/*2, *2/*17 and *17/*17 had representation of > 10% in our study population **(**Fig. 1**).**

**Table 1:** CYP2D6 genotypes and activity score

Diplotype	Number	Activity score^#^	Phenotype
*1/*1	15	2	NM (n = 76)
*1/*2	12		
*2/*2	9		
*1/*17	13	1.5	
*1/*29	2		
*2/*29	7		
*2/*17	11		
*1/*9	2		
*1/*10	2	1.25	
*2/*10	3		
*1/*4	3	1	IM (n = 28)
*1/*5	1		
*17/*17	8		
*17/*29	4		
*2/*3	1		
*2/*4	5		
*10/*17	3	0.75	
*10/*29	1		
*4/*29	1	0.5	
*4/*41	1		
*4/*4	2	0	PM (n = 2)

^#^Activity score was adapted from the *CYP2D6* Diplotype-Phenotype table at https://www.pharmgkb.org/page/cyp2d6RefMaterials by summing assigned values for each allele.

: Normal metabolizer IM: Intermediate metabolizer PM: Poor metabolizer

The frequency of the *CYP2D6* alleles *1, *2, *3, *4, *5, *9, *10, *17, *29 and *41 was 0.307, 0.269, 0.005, 0.066, 0.500, 0.009, 0.043,0.222,0.071 and 0.005 respectively (Supplementary Table S1). There were no CYP2D6 *1 × 2, *40, *43, *45, *106 and *149 found in our study population. Comparison of allele frequencies with what has previously been found in sub-Saharan populations also show the allele frequencies in our study population were comparable. However, alleles that were previously not discovered in our population such as *3, *9 and *41 were observed in our population.

**Table 2:** *CYP2D6* allelic frequencies in black African populations in sub–Saharan Africa in comparison to our study

Allele	CPIC phenotype	This study (n = 106)	GHA [22] (n = 26)	UGA #[23] (n = 99)	TZN [6] (n = 106)	GHA [24] (n = 193)	MDG [25] (n = 211)	ETH [26] (n = 81)	SA [27] (n = 100)	TZN [28] (n = 196)
*1	Normal	30.7	26.9	18.7	-	43.7	35.8	-	30.7	96.0
*2	Normal	26.9	13.5	25.8	-	10.6	6.4	33.3	4.5	-
*3	No function	0.5	-	-	-	-	-	-	-	-
*4	No function	6.6	-	3.5	1.0	6.3	2.1	4.9	2.3	4.0
*5	No function	0.5	-	9.6	6.0	6.0	1.7	4.3	5.7	-
*9	Decreased	0.9	-	-	-	-	-	-		-
*10	Decreased	4.3	-	1.5	-	3.1	17.1	1.9	6.8	-
*17	Decreased	22.2	21.2	19.2	17.0	27.7	10.9	10.5	13.6	-
*29	Decreased	7.1	5.8	13.1	-	-	-	-	-	-
*41	Decreased	0.5	-	4.0	-	-	3.6	-	-	-

^#^Allele frequencies were imputed from data provided

## Discussion

*CYP2D6* is one of the key pharmacogenes that is considered during opioid dosing according to the Clinical Pharmacogenetics Implementation Consortium (CPIC). This enzyme is significant in pain management regimen that include codeine, hydrocodone and tramadol therapy. Tramadol which is a pain medication for pre- and post-operative pain is highly influenced by *CYP2D6* variations [29]. The CYP2D6 enzyme is important because it changes tramadol into O-desmethyltramadol (M1), an active metabolite that has six times the analgesic ability of its parent drug, tramadol.

Our study showed that there was 71.7% representation of NMs in our study population whiles, 26.4% were IMs and 1.9% were PMs. Our data compares with predicted and observed phenotypes in other sub-Saharan African populations [22, 30] With this kind of representation, majority of individuals prescribed with tramadol should obtain analgesic effect once they go on the recommended age- and weight-specific dosage. On the other hand, though of a small proportion, individuals who are IMs may have a reduced analgesic effect because enough active metabolites are not being produced by CYP2D6. These patients therefore need alternative pharmacotherapy for their pain management. Essentially, such PMs, will not adequately benefit from the use of tramadol so they may have to go with an alternate medication.

From our comparative analysis with representative variants in other African populations, we found about 40% of the observed alleles in our study population had no and decreasing function (Table 2) and three alleles which had not been previously reported [22, 24] in any study involving Ghanaians, *3, *9 and *41 were observed. The no function CYP2D6*3 and decreased function CYP2D6*9 alleles which has previously not been reported in studies involving African populations (Table 2) is reported with a frequency of 0.5% and 0.9% in this study respectively. The frequency of CYP2D6*3, *9 and *41 is higher in individuals of European ancestry [31, 32] compared those of African ancestry. Our low frequency therefore is consistent with what is expected although our study population is low. CYP2D6*17 was the most frequent decreased function allele found in our study which is consistent with what has been observed generally among SSA population [33, 34] (Table 2). Over the past 5–7 years, there has been a tramadol ‘abuse crisis’ in Ghana especially among informal workers such as construction workers, illegal miners, drivers, slam dwellers, and high school students [35–38]. The question that arises is that “**is the so-called abuse emanating from some pharmacogenetic influence due to reduced or unresponsive analgesic effect or it is purposeful overdosing?**”

Having established that CYP2D6 is largely responsible for converting tramadol to its active form and the representation of IMs and PMs in our population ranges from 15 to 30% ,Table 1; Fig. 1 [22, 24], we could postulate that the ‘tramadol crisis’ could be as a result of individuals who obtain the tramadol from community pharmacies and over-the-counter medicine sellers who are not benefiting completely from the analgesic effects of tramadol and therefore self-overdosing based on the influence of testimonies from normal metabolisers. Although IMs are a less studied phenotype, they are at risk of decreased biotransformation of the CYP2D6-mediated tramadol to its potent metabolite M1 therefore conferring decreased analgesia [7].Our study showed a frequency of 26% IMs in our population and such individuals may have reduced benefits to tramadol. It has been shown in a previous study that IMs experience approximately 30% reduction in composite pain [39] in comparison NMs. Previous studies in Ghana on the ‘tramadol crisis’ found out the major reason for taking tramadol literally every day was to reduce pain so they can go about their activities immediately [20, 35] although others also took it for sexual, psychological and physical motivations [37].

This may mean that for those that are ‘abusing’ tramadol for pain relief such as construction workers, commercial drivers, illegal miners, if such individuals are IMs, they may tend to take a higher dosage with the hope of relieving their pain. This partial metabolism may come with adverse events and potentially the experiences of mood elevation, euphoria and false sense of happiness and strength [39, 40] such as that experienced with tramadol “abusers” in Ghana [20, 37]. If the tramadol were clinically prescribed, a clinician would have recommended an alternative per the CPIC guidelines and thus avoid the associated adverse effects.

There were few PMs in our study population who carry non-functional alleles representing approximately 2% (Table 1) agreeing with the estimated 0.4-5% across a population [3, 7]. Interestingly, tramadol “abusers” who are PMs will continue to overdose to feel the effect of the drug at the expense of tramadol-induced renal damage and neurotoxic outcomes including seizures [41, 42]. We are proposing that looking at the *CYP2D6* variability in our population from studies in our population, perhaps a look at pharmacogenetics in addition to other variables may also explain why the overdose and perhaps help with better approaches to curb the tramadol ‘abuse crisis’.

### Limitations to this study

The limitations in our study include the fact that our sample size is comparatively small (n = 106) and we did not have direct phenotype data. There could also be other undiscovered *CYP2D6* alleles in our population. Further studies that can make use of tramadol metabolites are therefore warranted in a relatively larger cohort in addition to exploring other *CYP2D6* alleles.

## Conclusion

Our study shows the wide variations in *CYP2D6* activity in addition to observing decreasing function alleles that were previously not reported in Ghanaians and other sub-Saharan African populations. Therefore, *CYP2D6* should be considered in opioid dosage while making use of these observed variations to implement new approaches to tackle the tramadol ‘abuse crisis’ in Ghana.

### Electronic supplementary material

Below is the link to the electronic supplementary material.

Supplementary Material 1

Supplementary Material 2

## Data Availability

The data for this study is presented within the article and supplementary data. The raw datasets generated during the study can be made available from the corresponding author on reasonable request. Raw data has been deposited in GEO submission with accession number GSE232865.

## Declarations

### Author information

N/A.
