# Supplementary material for: A consideration of CYP2D6 genetic variations in the Ghanaian population as a potential ‘culprit’ for the tramadol ‘abuse crisis’
Source: BMC Med Genomics. 2024 Jan 22;17:28. doi: 10.1186/s12920-023-01773-8 (PMC10804791; doi:10.1186/s12920-023-01773-8)
Supplement: Supplementary file 1 — Supplementary Material 1 [file 12920_2023_1773_MOESM1_ESM.docx]

**Supplementary Table S1: CYP2D6 genotype frequencies in the study population**

| **Variant** | **Frequency** |
| --- | --- |
| CYP2D6 rs16947  g.42127941G>A |  |
| A/A | 39 |
| G/A | 41 |
| G/G | 26 |
|  |  |
| CYP2D6 rs1135840  g.9200G>C |  |
| C/C | 20 |
| C/G | 32 |
| G/G | 54 |
|  |  |
| CYP2D6 rs35742686  g.7569del |  |
| T/T | 106 |
|  |  |
| CYP2D6 rs3892097  g.42128945C>T |  |
| C/C | 94 |
| C/T | 10 |
| T/T | 2 |
|  |  |
| CYP2D6 rs1065852  g.42130692G>A |  |
| A/A | 2 |
| A/G | 19 |
| G/G | 86 |
|  |  |
| CYP2D6 rs5030655  g.6727del |  |
| A/A | 106 |
|  |  |
| CYP2D6 rs5030867  g.42127856T>G |  |
| T/T | 106 |
|  |  |
| CYP2D6 rs5030865  g.42129033C>A |  |
| C/C | 106 |
|  |  |
| CYP2D6 rs28371706  g.42129770G>A |  |
| G/G | 65 |
| A/G | 33 |
| A/A | 8 |
|  |  |
| CYP2D6 rs59421388  g.42127608C>T |  |
| C/C | 91 |
| C/T | 15 |
|  |  |
| CYP2D6 rs28371725  g.42127803C>T |  |
| C/C | 104 |
| C/T | 1 |
| T/T | 1 |
